# Supplementary material for: Depth-dependent parental effects create invisible barriers to coral dispersal
Source: Commun Biol. 2021 Feb 15;4:202. doi: 10.1038/s42003-021-01727-9 (PMC7884412; doi:10.1038/s42003-021-01727-9)
Supplement: Supplementary file 2 — Description of Supplementary Files [file 42003_2021_1727_MOESM2_ESM.pdf]

Description of Additional Supplementary Files

File name: Supplementary Data 1

Description: Raw data of the different experiments and surveys performed in this study.
