# Supplementary material for: Depth-dependent parental effects create invisible barriers to coral dispersal
Source: Commun Biol. 2021 Feb 15;4:202. doi: 10.1038/s42003-021-01727-9 (PMC7884412; doi:10.1038/s42003-021-01727-9)
Supplement: Supplementary file 4 — Reporting Summary [file 42003_2021_1727_MOESM4_ESM.pdf]

nature research

Corresponding author(s): Tom Shlesinger

Last updated by author(s): Dec 18, 2020

Reporting Summary

Nature Research wishes to improve the reproducibility of the work that we publish. This form provides structure for consistency and transparency in reporting. For further information on Nature Research policies, see our Editorial Policies and the Editorial Policy Checklist.

Statistics

For all statistical analyses, confirm that the following items are present in the figure legend, table legend, main text, or Methods section.

n/a	Confirmed	
☐	☒	The exact sample size (*n*) for each experimental group/condition, given as a discrete number and unit of measurement
☐	☒	A statement on whether measurements were taken from distinct samples or whether the same sample was measured repeatedly
☐	☒	The statistical test(s) used AND whether they are one- or two-sided<br>*Only common tests should be described solely by name; describe more complex techniques in the Methods section.*
☐	☒	A description of all covariates tested
☐	☒	A description of any assumptions or corrections, such as tests of normality and adjustment for multiple comparisons
☐	☒	A full description of the statistical parameters including central tendency (e.g. means) or other basic estimates (e.g. regression coefficient) AND variation (e.g. standard deviation) or associated estimates of uncertainty (e.g. confidence intervals)
☐	☒	For null hypothesis testing, the test statistic (e.g. *F*, *t*, *r*) with confidence intervals, effect sizes, degrees of freedom and *P* value noted<br>*Give P values as exact values whenever suitable.*
☒	☐	For Bayesian analysis, information on the choice of priors and Markov chain Monte Carlo settings
☒	☐	For hierarchical and complex designs, identification of the appropriate level for tests and full reporting of outcomes
☐	☒	Estimates of effect sizes (e.g. Cohen's *d*, Pearson's *r*), indicating how they were calculated

Our web collection on statistics for biologists contains articles on many of the points above.

Software and code

Policy information about availability of computer code

Data collection	No software was used.
Data analysis	R v3.6.1: A language and environment for statistical computing.

For manuscripts utilizing custom algorithms or software that are central to the research but not yet described in published literature, software must be made available to editors and reviewers. We strongly encourage code deposition in a community repository (e.g. GitHub). See the Nature Research guidelines for submitting code & software for further information.

Data

Policy information about availability of data

All manuscripts must include a data availability statement. This statement should provide the following information, where applicable:

- Accession codes, unique identifiers, or web links for publicly available datasets
- A list of figures that have associated raw data
- A description of any restrictions on data availability

All data generated and analyzed during this study are included in a supplementary data file.

Field-specific reporting

Ecological, evolutionary & environmental sciences study design

All studies must disclose on these points even when the disclosure is negative.

Study description	This study included experiments with coral larvae of three species, as well as ecological field survey at two sites in different depths. For the field survey, 50 photo-quadrats were analyzed in each site and depth (a total of 700 photo-quadrats). In the different experiments, larvae were collected and pooled from 5-10 coral colonies and were then randomly divided among several treatment groups. The two treatment factors were light regime and settlement tiles conditioned in different depths. In all experiments, each treatment group comprised 5-6 replicates, each containing 30-40 planulae.
Research sample	For each of the studied species, adult corals and their larvae were haphazardly sampled from two distinct depths. Sampling from the two depths represented two populations of a given species: a shallow-water (5 m depth) population and a mesophotic (45 m depth) population.
Sampling strategy	As the coral species we studied release dozens to hundreds of larvae every day during their reproductive period, five colonies were proven to be suffice in order to get enough larvae needed to perform our planned experiments. In the settlement experiments, we did not use any statistical method to predetermine the sample size and we followed standards and number of replications/larvae used in numerous earlier marine larvae settlement studies.
Data collection	For the field surveys, we photographed dozens of quadrats in each site and in each depth and then recorded the number of colonies of each species in each quadrat. In the settlement experiments, we performed daily counts of settled larvae (with the aid of magnifying glass) along a period of ten days. In the survival experiments, we counted the number of surviving juvenile corals in three-week intervals along three months and in the end of the experiment period we measured the surviving corals' size using a microscope equipped with a camera.
Timing and spatial scale	Surveys were performed at seven depth stations (2, 5, 10, 20, 30, 40, and 50 m) on two reefs separated by a few hundred meters at the northern Gulf of Eilat/Aqaba, Red Sea (29°30'08.5"N 34°55'07.7"E).<br>For the settlement experiments, corals (or larvae) were collected during the expected peak reproductive season of each species (based on previous studies) to assure that their larvae release will be maximized. Corals and larvae were collected from two contrasting depths: from a shallow reef (ca. 5 m) and from a mesophotic reef (ca. 45 m). The settlement experiments were initiated immediately upon the collection of larvae from the collected corals and we counted the number of settled larvae on a daily basis along a period of ten days. In all of our experiments, the vast majority of larvae settled within the first few days and only few of them remained as free-swimming after a week. Therefore, ten-day periods for these experiments were found to be suitable as in most of our treatments there were none to very few free-swimming larvae at the end of the experiment, i.e., after ten days.
Data exclusions	No data were excluded from the analyses
Reproducibility	We conducted our experiments with several species and two populations separated by distinct depths to strengthen any possible finding we would have. Every experiment included 5 or 6 replicates and, generally, all experimental attempts were successful.
Randomization	All collected larvae originated from 5 to 10 different parental colonies for each species in each depth. Following the collection of the larvae from several colonies of each species and depth, they were pooled in one aquarium and then randomly distributed among the different treatment groups.
Blinding	Blinding was not used during data acquisition and analysis. All analyzed data are presented in this study without exclusion.

Did the study involve field work? ☒ Yes ☐ No

Field work, collection and transport

Field conditions	Sea conditions at the study location (i.e., the Gulf of Eilat/Aqaba, northern Red Sea) are rather calm and stable, usually with only mild winds and currents, and sea water temperatures that range between 21 and 29 degrees Celsius.
Location	Coral collections and surveys were performed on a near shore, fringing reef in the Gulf of Eilat and Aqaba, Red Sea (29°30'08.5"N 34° 55'07.7"E), at a depth of ca. 2 - 50 m.
Access & import/export	Corals were collected and surveyed under the permits of the Israeli Nature and Park Authority provided to the authors on February 05, 2018 (permit numbers 2018/41871 and 2018/41878) and on May 6, 2018 (permit number 2018/41953). The surveyed reefs and collected corals were accessed from shore by either recreational diving or technical diving using close-circuit rebreathers. Access, collections, and field work were conducted in compliance with local, national, and international laws.
Disturbance	Disturbance was minimized by collecting only a relatively small number of corals, which were sufficient for this study, while carefully avoiding any physical contact and damage to the surrounding reef and other organisms while sampling. Following the collection of coral larvae in the aquaria, the coral colonies were returned to their native depth on the reef.

Reporting for specific materials, systems and methods

We require information from authors about some types of materials, experimental systems and methods used in many studies. Here, indicate whether each material, system or method listed is relevant to your study. If you are not sure if a list item applies to your research, read the appropriate section before selecting a response.

Materials & experimental systems

n/a	Involved in the study
☒	☐ Antibodies
☒	☐ Eukaryotic cell lines
☒	☐ Palaeontology and archaeology
☐	☒ Animals and other organisms
☒	☐ Human research participants
☒	☐ Clinical data
☒	☐ Dual use research of concern

Methods

n/a	Involved in the study
☒	☐ ChIP-seq
☒	☐ Flow cytometry
☒	☐ MRI-based neuroimaging

Animals and other organisms

Policy information about studies involving animals; ARRIVE guidelines recommended for reporting animal research

Laboratory animals	This study did not involve laboratory animals.
Wild animals	Wild corals of two Stylophora species were collected by gently dislocating them from the reef and transferring them to the experimental aquaria. These were hermaphroditic species that were used to collect larvae for the experiments. Additional coral larvae were directly collected from female colonies of another species (Rhytisma sp.) during their surface-brooding period, thus avoiding collection of the corals themselves. At the end of all larvae extractions (i.e., within 2 to 14 days), all corals were placed back on the reef in their natal environment.
Field-collected samples	Collected corals were placed in experimental aquaria supplied with continuous flow of seawater (i.e., an open-circulation system) from ca. 40 m depth and were maintained in the best possible conditions.
Ethics oversight	As corals are marine invertebrates, they do not yet have ethical oversight. Collection procedures were approved by the Israel Nature and Park Authority.

Note that full information on the approval of the study protocol must also be provided in the manuscript.
